# Review: Diversity of Microorganisms in Global Fermented Foods and Beverages

**DOI:** 10.3389/fmicb.2016.00377

**Published:** 2016-03-24

**Authors:** Jyoti P. Tamang, Koichi Watanabe, Wilhelm H. Holzapfel

**Affiliations:** ^1^Department of Microbiology, School of Life Sciences, Sikkim UniversityTadong, India; ^2^Department of Animal Science and Technology, National Taiwan UniversityTaipei, Taiwan; ^3^Advance Green Energy and Environment Institute, Handong Global UniversityPohang-si, South Korea

**Keywords:** global fermented foods, LAB, *Bacillus*, yeasts, filamentous molds

## Abstract

Culturalable and non-culturable microorganisms naturally ferment majority of global fermented foods and beverages. Traditional food fermentation represents an extremely valuable cultural heritage in most regions, and harbors a huge genetic potential of valuable but hitherto undiscovered strains. Holistic approaches for identification and complete profiling of both culturalable and non-culturable microorganisms in global fermented foods are of interest to food microbiologists. The application of culture-independent technique has thrown new light on the diversity of a number of hitherto unknown and non-cultural microorganisms in naturally fermented foods. Functional bacterial groups (“phylotypes”) may be reflected by their mRNA expression in a particular substrate and not by mere DNA-level detection. An attempt has been made to review the microbiology of some fermented foods and alcoholic beverages of the world.

## Introduction

Traditionally, boiled rice is a staple diet with fermented and non-fermented legume (mostly soybeans) products, vegetables, pickles, fish, and meat in Far-East Asia, South Asia, North Asia, and the Indian subcontinent excluding Western and Northern India; while wheat/barley-based breads/loaves comprise a staple diet followed by milk and fermented milk products, meat, and fermented meats (sausages) in the Western and Northern part of India, West Asian continent, Europe, North America, and even in Australia and New Zealand (Tamang and Samuel, 2010). Sorghum/maize porridges, on the other hand, are the main courses of diet with many fermented and non-fermented sorghum/maize/millets, cassava, wild legume seeds, meat, and milk products in Africa and South America. Fermented foods are the hub of consortia of microorganisms, since they are either present as natural indigenous microbiota in uncooked plant or animal substrates, utensils, containers, earthen pots, and the environment (Hesseltine, 1979; Franz et al., 2014), or add starter culture(s) containing functional microorganisms (Holzapfel, 1997; Stevens and Nabors, 2009) which modify the substrates biochemically, and organoleptically into edible products that are culturally and socially acceptable to the consumers (Campbell-Platt, 1994; Steinkraus, 1997; Tamang, 2010b). Microorganisms convert the chemical composition of raw materials during fermentation, which enrich the nutritional value in some fermented foods, and impart health-benefits to the consumers (Steinkraus, 2002; Farhad et al., 2010; Tamang, 2015a).

Several researchers have reviewed the microbiology, biochemistry, and nutrition of fermented foods and beverages from different countries of Asia (Hesseltine, 1983; Steinkraus, 1994, 1996; Nout and Aidoo, 2002; Tamang et al., 2015); Africa (Odunfa and Oyewole, 1997; Olasupo et al., 2010; Franz et al., 2014); Europe (Pederson, 1979; Campbell-Platt, 1987; Wood, 1998); South America (Chaves-López et al., 2014), and North America (Doyle and Beuchat, 2013). Many genera/species of microorganisms have been reported in relation to various fermented foods and beverages across the world; the usage of molecular tools in recent years have helped to clarify, at least in part, the nomenclatural confusion and generalization caused by conventional (phenotypic) taxonomic methods. The present paper is an attempt to collate and review the updated information on microbiology of some globally fermented foods and beverages.

### Microorganisms in fermented foods

Lactic acid bacteria (LAB) are widely present in many fermented foods and beverages (Stiles and Holzapfel, 1997; Tamang, 2010b). Major genera of the LAB such as *Alkalibacterium, Carnobacterium, Enterococcus, Lactobacillus, Lactococcus, Leuconostoc, Oenococcus, Pediococcus, Streptococcus, Tetragenococcus, Vagococcus*, and *Weissella* (Salminen et al., 2004; Axelsson et al., 2012; Holzapfel and Wood, 2014) have been isolated from various globally fermented foods and beverages.

*Bacillus* is present in alkaline-fermented foods of Asia and Africa (Parkouda et al., 2009; Tamang, 2015b). Species of *Bacillus* that are present, mostly in legume-based fermented foods, are *Bacillus amyloliquefaciens, Bacillus circulans, Bacillus coagulans, Bacillus firmus, Bacillus licheniformis, Bacillus megaterium, Bacillus pumilus, Bacillus subtilis, Bacillus subtilis* variety *natto*, and *Bacillus thuringiensis* (Kiers et al., 2000; Kubo et al., 2011), while strains of *Bacillus cereus* have been isolated from the fermentation of *Prosopis africana* seeds for the production of *okpehe* in Nigeria (Oguntoyinbo et al., 2007). Some strains of *B*. *subtilis* produce λ-polyglutamic acid (PGA) which is an amino acid polymer commonly present in Asian fermented soybean foods, giving the characteristic of a sticky texture to the product (Urushibata et al., 2002; Nishito et al., 2010).

The association of several species of *Kocuria, Micrococcus* (members of the *Actinobacteria*), and *Staphylococcus* (belonging to the *Firmicutes*) has been reported for fermented milk products, fermented sausages, meat, and fish products (Martín et al., 2006; Coton et al., 2010). Species of *Bifidobacterium, Brachybacterium, Brevibacterium*, and *Propionibacterium* are isolated from cheese, and species of *Arthrobacter* and *Hafnia* from fermented meat products (Bourdichon et al., 2012). *Enterobacter cloacae, Klebsiella pneumoniae, K. pneumoniae* subsp. *ozaenae, Haloanaerobium, Halobacterium, Halococcus, Propionibacterium, Pseudomonas*, etc. are also present in many global fermented foods (Tamang, 2010b).

Genera of yeasts reported for fermented foods, alcoholic beverages and non-food mixed amylolytic starters are mostly *Brettanomyces, Candida, Cryptococcus, Debaryomyces, Dekkera, Galactomyces, Geotrichum, Hansenula, Hanseniaspora, Hyphopichia, Issatchenkia, Kazachstania, Kluyveromyces, Metschnikowia, Pichia, Rhodotorula, Rhodosporidium, Saccharomyces, Saccharomycodes, Saccharomycopsis, Schizosaccharomyces, Sporobolomyces, Torulaspora, Torulopsis, Trichosporon, Yarrowia*, and *Zygosaccharomyces* (Watanabe et al., 2008; Tamang and Fleet, 2009; Lv et al., 2013).

Major role of filamentous molds in fermented foods and alcoholic beverages is the production of enzymes and the degradation of anti-nutritive factors (Aidoo and Nout, 2010). Species of *Actinomucor, Amylomyces, Aspergillus, Monascus, Mucor, Neurospora, Parcilomyces, Penicillium, Rhizopus*, and *Ustilago* are reported for many fermented foods, Asian non-food amylolytic starters and alcoholic beverages (Nout and Aidoo, 2002; Chen et al., 2014).

## Taxonomic tools for identification of microorganisms from fermented foods

Use of culture media may ignore several unknown non-culturable microorganisms that may play major or minor functional roles in production of fermented foods. Direct DNA extraction from samples of fermented foods, commonly known as culture-independent methods, is nowadays frequently used in food microbiology to profile both culturable and non-culturable microbial populations from fermented foods (Cocolin and Ercolini, 2008; Alegría et al., 2011; Cocolin et al., 2013; Dolci et al., 2015), provided that the amplification efficiency is high enough. PCR-DGGE analysis is the most popular culture-independent technique used for detecting microorganisms in fermented foods and thereby profiling both bacterial populations (Cocolin et al., 2011; Tamang, 2014) and yeast populations in fermented foods (Cocolin et al., 2002; Jianzhonga et al., 2009). Both culturable and non-culturable microorganisms from any fermented food and beverage may be identified using culture-dependent and -independent methods to document a complete profile of microorganisms, and also to study both inter- and intra-species diversity within a particular genus or among genera (Ramos et al., 2010; Greppi et al., 2013a,b; Yan et al., 2013). A combination of Propidium MonoAzide (PMA) treatment on samples before DNA extraction and molecular quantifying method can be used to accurately enumerate the viable microorganisms in fermented foods (Desfossés-Foucault et al., 2012; Fujimoto and Watanabe, 2013).

Molecular identification is emerging as an accurate and reliable identification tool, and is widely used in identification of both culture-dependent and culture-independent microorganisms from fermented foods (Giraffa and Carminati, 2008; Dolci et al., 2015). Species-specific PCR primers are used for species level identification (Tamang et al., 2005); this technique is widely applied in the identification of LAB isolated from fermented foods (Robert et al., 2009). The application of real-time quantitative PCR (qPCR) with specific primers enables the specific detection and quantification of LAB species in fermented foods (Park et al., 2009).

Random amplification of polymorphic DNA (RAPD) is a typing method based on the genomic DNA fragment profiles amplified by PCR, and is commonly used for disintegration of LAB strains from fermented foods (Coppola et al., 2006; Chao et al., 2008). The repetitive extragenic palindromic sequence-based PCR (rep-PCR) technique permits typing at subspecies level and reveals significant genotypic differences among strains of the same bacterial species from fermented food samples (Tamang et al., 2008). Amplified fragment length polymorphism (AFLP) is a technique based on the selective amplification and separation of genomic restriction fragments, and its applicability in identification and to discriminate has been demonstrated for various LAB strains (Tanigawa and Watanabe, 2011).

Techniques of denaturing gradient gel electrophoresis (DGGE) and temperature gradient gel electrophoresis (TGGE) have been developed to profile microbial communities directly from fermented foods, and are based on sequence-specific distinctions of 16S rDNA and 26S rDNA amplicons produced by PCR (Ercolini, 2004; Flórez and Mayo, 2006; Alegría et al., 2011). However, DGGE has some disadvantages as well like it is time consuming, unable to determine the relative abundance of dominant species and distinguish between viable and nonviable cells, as well as it has difficulties in interpretation of multi-bands (Dolci et al., 2015). DGGE is also limited to detect specific species as it may only reveal some of the major bacterial species such as *B. licheniformis* and *Bacillus thermoamylovorans* in *chungkokjang* (sticky fermented soybean food of Korea) and not detect a large number of predominant or diverse rare bacterial species identified in pyrosequencing analysis (Nam et al., 2011).

The amplified ribosomal DNA restriction analysis (ARDRA) technique using restriction enzymes is also useful in identification of microorganisms from fermented foods (Jeyaram et al., 2010).

Multilocus sequence analysis (MLSA), using housekeeping genes as molecular markers alternative to the 16S rRNA genes, is used for LAB species identification: *rpoA* and *pheS* genes for *Enterococcus* and *Lactobacillus, atpA* and *pepN* for *Lactococcus* species, and *dnaA, gyrB*, and *rpoC* for species of *Leuconostoc, Oenococcus*, and *Weissella* (de Bruyne et al., 2007, 2008b, 2010; Diancourt et al., 2007; Picozzi et al., 2010; Tanigawa and Watanabe, 2011).

Effective tools of next generation sequencing (NGS) such as metagenomics, phylobiomics, and metatranscriptomics are nowadays applied for documentation of cultures in traditionally fermented products (Mozzi et al., 2013; van Hijum et al., 2013). However, NGS as a sophisticated tool needs well-trained hands and a well-equipped molecular laboratory, which may not always be available. Application of metagenomic approaches, by using parallel pyrosequencing of tagged 16S rRNA gene amplicons, provide information on microbial communities as profiled in *kimchi*, a naturally fermented vegetable product of Korea (Jung et al., 2011; Park et al., 2012), *nukadoko*, a fermented rice bran of Japan (Sakamoto et al., 2011), *narezushi*, a fermented salted fish and cooked rice of Japan (Kiyohara et al., 2012), and *ben-saalga*, a traditional gruel of pearl millet of Burkina Faso (Humblot and Guyot, 2009). Pyrosequencing has revealed the presence of numerous and even minor bacterial groups in fermented foods, but DNA-level detection does not distinguish between metabolically “active” and “passive” organisms. “Functionally relevant phylotypes” in an ecosystem may be specifically detected by, e.g., weighted UniFrac principal coordinate analysis based on 454 pyrosequencing of 16S rRNA genes, as applied in studies on gut microbiota (Wang et al., 2015). The 16S rRNA gene sequence based pyrosequencing method enables a comprehensive and high-throughput analysis of microbial ecology (Sakamoto et al., 2011), and this method has been applied to various traditionally fermented foods (Oki et al., 2014).

A proteomics identification method based on protein profiling using matrix-assisted laser desorption ionizing-time of flight mass spectrometry (MALDI-TOF MS) has been used to identify species of *Bacillus* in fermented foods of Africa (Savadogo et al., 2011), and species of LAB isolated from global fermented foods (Tanigawa et al., 2010; Dušková et al., 2012; Sato et al., 2012; Nguyen et al., 2013a; Kuda et al., 2014).

### Global fermented foods

Campbell-Platt (1987) reported around 3500 global fermented foods and beverages, and had divided them into about 250 groups. There might be more than 5000 varieties of common and uncommon fermented foods and alcoholic beverages being consumed in the world today by billions of people, as staple and other food components (Tamang, 2010b). Global fermented foods are classified into nine major groups on the basis of substrates (raw materials) used from plant/animal sources: (1) fermented cereals, (2) fermented vegetables and bamboo shoots, (3) fermented legumes, (4) fermented roots/tubers, (5) fermented milk products, (6) fermented and preserved meat products, (7) fermented, dried and smoked fish products, (8) miscellaneous fermented products, and (9) alcoholic beverages (Steinkraus, 1997; Tamang, 2010b,c).

### Fermented milk products

Fermented milk products (Table 1) are classified into two major groups on the basis of microorganisms: (A) lactic fermentation, dominated by species of LAB, comprising the “thermophilic” type (e.g., yogurt, Bulgarian buttermilk), probiotic type (e.g., acidophilus milk, bifidus milk), and the mesophilic type (e.g., natural fermented milk, cultured milk, cultured cream, cultured buttermilk); and (B) fungal-lactic fermentations, where LAB and yeasts cooperate to generate the final product, which include alcoholic milks (e.g., acidophilus-yeast milk, *kefir, koumiss*), and moldy milks (e.g., *viili*; Mayo et al., 2010). Natural fermentation is one of the oldest methods of milk processing using raw and boiled milk to ferment spontaneously, or of using the back-slopping method where a part of the previous batch of a fermented product is used to inoculate the new batch (Holzapfel, 2002; Josephsen and Jespersen, 2004). Cheese and cheese products derived from the fermentation of milk are of major nutritional and commercial importance throughout the world (de Ramesh et al., 2006). Starter cultures in milk fermentation are of two types: primary cultures that are mostly *Lactococcus lactis* subsp. *cremoris, Lc. lactis* subsp. *lactis, Lactobacillus delbrueckii* subsp. *delbrueckii, Lb. delbrueckii* subsp. *lactis, Lb. helveticus, Leuconostoc* spp., and *Streptococcus thermophilus* to participate in the acidification (Parente and Cogan, 2004); and secondary cultures that are used in cheese-making are *Brevibacterium linens, Propionibacterium freudenreichii, Debaryomyces hansenii, Geotrichum candidum, Penicillium camemberti*, and *P. roqueforti* for development of flavor and texture during ripening of cheese (Coppola et al., 2006; Quigley et al., 2011). Some non-starter lactic acid bacteria (NSLAB) microbiota are usually present in high numbers in fermented milk, which include *Enterococcus durans, Ent. faecium, Lb. casei, Lb. plantarum, Lb. salivarius*, and *Staphylococcus* spp. (Briggiler-Marcó et al., 2007).

**Table 1:** **Microorganisms isolated from some common and uncommon fermented milk products of the world**.

**Product**	**Substrate**	**Sensory property and nature**	**Microorganisms**	**Country**	**References**
*Airag*	Mare or camel milk	Acidic, sour, mild alcoholic drink	*Lb. helveticus, Lb. kefiranofaciens, Bifidobacterium mongoliense, Kluyveromyces marxianus*	Mongolia	Watanabe et al., 2008, 2009b; Yu et al., 2011
*Amasi*	Cow milk	Acidic, sour, with thick consistency	*Lc. lactis* subsp. *lactis* (dominating), *Lc. lactis* subsp. *cremoris, Lactobacillus, Enterococcus*, and *Leuconostoc* spp. Several non-culturable strains	South Africa, Zimbabwe	Osvik et al., 2013
*Cheese*	Animal milk	Soft or hard, solid; side dish, salad, used in many cooked/baked dishes	*Lc. lactis* subsp. *cremoris, Lc. lactis* subsp. *lactis, Lb. delbrueckii* subsp. *delbrueckii, Lb. delbrueckii* subsp. *lactis, Lb. helveticus, Lb. casei, Lb. plantarum, Lb. salivarius, Leuconostoc* spp., *Strep. thermophilus, Ent. durans, Ent. faecium*, and *Staphylococcus* spp., *Brevibacterium linens, Propionibacterium freudenreichii, Debaryomyces hansenii, Geotrichum candidum, Penicillium camemberti, P. roqueforti*	Worldwide	Parente and Cogan, 2004; Quigley et al., 2011
*Chhu*	Yak/cow milk	Cheese like product, curry, soup	*Lb. farciminis, Lb. brevis, Lb. alimentarius, Lb. salivarius, Lact. lactis, Candida* sp. *Saccharomycopsis* sp.	India, Nepal, Bhutan, China (Tibet)	Dewan and Tamang, 2006
*Chhurpi*	Yak/cow milk	Cheese like product, soup, curry, pickle	*Lb. farciminis, Lb. paracasei, Lb. biofermentans, Lb. plantarum, Lb. curvatus, Lb. fermentum, Lb. alimentarius, Lb. kefir, Lb. hilgardii, W. confusa, Ent. faecium, Leuc. mesenteroides*	India, Nepal, Bhutan, China (Tibet)	Tamang et al., 2000
*Dahi*	Cow/buffalo milk, starter culture	Curd, savory	*Lb. bifermentans, Lb. alimentarius, Lb. paracasei, Lact. lactis, Strep. cremoris, Strep. lactis, Strep. thermophilus, Lb. bulgaricus, Lb. acidophilus, Lb. helveticus, Lb. cremoris, Ped. pentosaceous, P. acidilactici, W. cibaria, W. paramesenteroides, Lb. fermentum, Lb. delbrueckii* subsp. *indicus, Saccharomycopsis* sp.*, Candida* sp.	India, Nepal, Sri Lanka, Bangladesh, Pakistan	Harun-ur-Rashid et al., 2007; Patil et al., 2010
*Dadih*	Buffalo milk	Curd, savory	*Leuc. mesenteroides, Ent. faecalis, Strep. lactis* supsp. *lactis, Strep. cremoris, Lb. casei* subsp. *casei*, and *Lb. casei* subsp. *rhamnosus*	Indonesia	Hosono et al., 1989
*Kefir*	Goat, sheep, cow	Alcoholic fermented milk, effervescent milk	*Lb. brevis, Lb. caucasicus, Strep. thermophilus, Lb. bulgaricus, Lb. plantarum, Lb. casei, Lb. brevis, Tor. holmii, Tor. delbruechii*	Russia	Bernardeau et al., 2006
*Koumiss*	Milk	Acid fermented milk, drink	*Lb. bulgaricus, Lb. salivarius, Lb. buchneri, Lb. heveticus, Lb. plantarum, Lb. acidophilus, Torula* sp.	Russia, Mongolia	Wu et al., 2009; Hao et al., 2010
*Laban rayeb*	Milk	Acid fermented milk, yogurt-like	*Lb. casei, Lb. plantarum, Lb. brevis, Lact. lactis, Leuconostoc* sp., *Sacch. kefir*	Egypt	Bernardeau et al., 2006
*Leben / Lben*	Cow milk	Sour milk	*Candida* sp., *Saccharomyces* sp., *Lactobacillus* sp., *Leuconostoc* sp.	North, East Central Africa	Odunfa and Oyewole, 1997
*Misti dahi (mishti doi, lal dahi, payodhi)*	Buffalo/cow milk	Mild-acidic, thick-gel, sweetened curd, savory	*Strep. Salivarius* subsp. *thermophilus, Lb. acidophilus, Lb. delbrueckii* subsp. *bulgaricus, Lc. lactis* subsp. *lactis, Sacch. cerevisiae*	India, Bangladesh	Ghosh and Rajorhia, 1990; Gupta et al., 2000
*Nunu*	Raw cow milk	Naturally fermented milk	*Lb. fermentum, Lb. plantarum, Lb. helveticus, Leuc. mesenteroides, Ent. faecium, Ent. italicus, Weissella confusa, Candida parapsilosis, C. rugosa, C. tropicalis, Galactomyces geotrichum, Pichia kudriavzevii, Sacch. cerevisiae*	Ghana	Akabanda et al., 2013
*Philu*	Cow/ yak milk	Cream like product, curry	*Lb. paracasei, Lb. bifermentans, Ent. faecium*	India, Nepal, Tibet (China)	Dewan and Tamang, 2007
*Shrikhand*	Cow, buffalo milk	Acidic, concentrated sweetened viscous, savory	*Lc. lactis* subsp. *lactis, Lc. lactis* subsp. *diacetylactis, Lc. lactis* subsp. *cremoris, Strep. thermophilus, Lb. delbruecki* subsp. *bulgaricus*	India	Sarkar, 2008; Singh and Singh, 2014
*Somar*	Yak or cow milk	Buttermilk	*Lb. paracasei, Lact. lactis*	India, Nepal	Dewan and Tamang, 2007
*Sua chua*	Dried skim milk, starter, sugar	Acid fermented milk	*Lb. bulgaricus, Strep. thermophilus*	Vietnam	Alexandraki et al., 2013
*Tarag*	Cow/yak/goat milk	Acidic, sour, drink	*Lb. delbrueckii* subsp. *bulgaricus, Lb. helveticus, Strep. thermophilus, Sacch. cerevisiae, Issatchenkia orientalis, Kazachstania unispora*	Mongolia	Watanabe et al., 2008
*Viili*	Cow milk	Thick and sticky, sweet taste, breakfast	*Lc. lactis* subsp. *lactis, Lc. lactis* subsp. *cremoris, Lc. lactis* subsp. *lactis* biovar. *diacetylactis, Leuc. mesenteroides* subps. *cremoris, G. candidum, K. marxianus, P. fermentans*	Finland	Kahala et al., 2008
Yogurt	Animal milk	Acidic, thick-gel viscous, Curd-like product, savory	*Strep. thermophilus, Lb. delbrueckii* subsp. *bulgaricus, Lb. acidophilus, Lb. casei, Lb. rhamnosus, Lb. gasseri, Lb. johnsonii, Bifidobacterium* spp.	Europe, Australia, America	Tamime and Robinson, 2007; Angelakis et al., 2011

### Fermented cereal foods

In most of the Asian countries, rice is fermented either by using mixed-culture(s) into alcoholic beverages or by using food beverages (Tamang, 2010c), whereas in Europe, America, and Australia, most cereals like wheat, rye, barley and maize are fermented by natural fermentation or by adding commercial baker's yeast into the batter for dough breads/loaves (Guyot, 2010). In Africa, fermented cereal foods are traditionally used as staples as well as complementary and weaning foods for infants and young children (Tou et al., 2007). In Europe, people still practice the old traditional method of preparation of breads or loaves without using any commercial strains of baker's yeast (Hammes and Ganzle, 1998). Yeasts and LAB conduct dough fermentation, mostly San Francisco sourdough, and the resultant products are generally called sourdough breads because they have higher contents of lactic acid and acetic acid due to the bacterial growth (Brandt, 2007; de Vuyst et al., 2009).

Cereal fermentation is mainly represented by species of LAB and yeasts (Corsetti and Settanni, 2007). *Enterococcus, Lactococcus, Lactobacillus, Leuconostoc, Pediococcus, Streptococcus*, and *Weissella* are common bacteria associated with cereal fermentations (Table 2; de Vuyst et al., 2009; Guyot, 2010; Moroni et al., 2011). Native strains of *Saccharomyces cerevisiae* are the principal yeast of most bread fermentations (Hammes et al., 2005), but other non-*Saccharomyces* yeasts are also significant in many cereal fermentations including *Candida, Debaryomyces, Hansenula, Kazachstania, Pichia, Trichosporon*, and *Yarrowia* (Iacumin et al., 2009; Weckx et al., 2010; Johnson and Echavarri-Erasun, 2011).

**Table 2:** **Microorganisms isolated from some common and uncommon fermented cereal foods of the world**.

**Product**	**Raw material/Substrate**	**Sensory property and nature**	**Microorganisms**	**Country**	**References**
*Ang-kak*	Red rice	Colorant	*Monascus purpureus*	China, Taiwan, Thailand, Philippines	Steinkraus, 1996
*Boza*	Cereals	Sour refreshing liquid	*Lactobacillus* sp., *Lactococcus* sp., *Pediococcus* sp., *Leuconostoc* sp., *Sacch. cerevisiae*	Bulgaria	Blandino et al., 2003
*Busa*	Maize, sorghum, millet	Submerged	*Sacch. cerevisiae, Schizosacchromyces pombe, Lb. plantarum, Lb. helveticus, Lb. salivarius, Lb. casei, Lb. brevis, Lb. buchneri, Leuc. mesenteroides, Ped. damnosus*	East Africa, Kenya	Odunfa and Oyewole, 1997
*Ben-saalga*	Pearl millet	Weaning food	*Lactobacillus* sp.*, Pediococcus* sp.*, Leuconostoc* sp.*, Weissela* sp., yeasts	Burkina Faso, Ghana	Tou et al., 2007
*Dosa*	Rice and black gram	Thin, crisp pancake, Shallow-fried, staple	*Leuc. mesenteroides, Ent. faecalis, Tor. candida, Trichosporon pullulans*	India, Sri Lanka, Malaysia, Singapore	Soni et al., 1986
*Enjera/Injera*	Tef flour, wheat	Acidic, sourdough, leavened, pancake-like bread, staple	*Lb. pontis, Lb. plantarum, Leuc. mesenteroides, Ped. cerevisiae, Sacch. cerevisiae, Cand. glabrata*	Ethiopia	Olasupo et al., 2010
*Gowé*	Maize	Intermediate product used to prepare beverages, porridges	*Lb. fermentum, Lb. reuteri, Lb. brevis, Lb. confusus, Lb. curvatus, Lb. buchneri, Lb. salivarius, Lact. lactis, Ped. pentosaceus, Ped. acidilactici, Leuc. mesenteroides; Candidatropicalis, C. krusei, Kluyveromyces marxianus*	Benin	Vieira-Dalodé et al., 2007; Greppi et al., 2013a
*Hussuwa*	Sorghum	Cooked dough	*Lb. fermentum, Ped. acidilactici, Ped. pentosaceus*, Yeasts	Sudan	Yousif et al., 2010
*Idli*	Rice, black gram or other dehusked pulses	Mild-acidic, soft, moist, spongy pudding; staple, breakfast	*Leuc. mesenteroides, Lb. delbrueckii, Lb. fermenti, Lb. coryniformis, Ped. acidilactis, Ped. cerevisae, Streptococcus* sp.*, Ent. faecalis, Lact. lactis, B. amyloliquefaciens, Cand. cacaoi, Cand. fragicola, Cand. glabrata, Cand. kefyr, Cand. pseudotropicalis, Cand. sake, Cand. tropicalis, Deb. hansenii, Deb. tamarii, Issatchenkia terricola, Rhiz. graminis, Sacch. cerevisiae, Tor. candida, Tor. holmii*	India, Sri Lanka, Malaysia, Singapore	Steinkraus et al., 1967; Sridevi et al., 2010
*Jalebi*	Wheat flour	Crispy sweet, doughnut-like, deep-fried, snacks	*Sacch. Bayanus, Lb. fermentum, Lb. buchneri, Lact. lactis, Ent. faecalis, Sacch. cerevisiae*	India, Nepal, Pakistan	Batra and Millner, 1976
*Kenkey*	Maize	Acidic, solid, steamed dumpling, staple	*Lb. plantarum, Lb. brevis, Ent. cloacae, Acinetobacter* sp., *Sacch. cerevisiae, Cand. mycoderma*	Ghana	Oguntoyinbo et al., 2011
*Khamak (Kao-mak)*	Glutinous rice, *Look-pang* (starter)	Dessert	*Rhizopus* sp.*, Mucor* sp.*, Penicillum* sp.*, Aspergillus* sp.*, Endomycopsis* sp.*, Hansenula* sp.*, Saccharomyces* sp.	Thailand	Alexandraki et al., 2013
*Kunu-zaki*	Maize, sorghum, millet	Mild-acidic, viscous, porridge, staple	*Lb. plantarum, Lb. pantheris, Lb. vaccinostercus, Corynebacterium* sp.*, Aerobacter sp., Cand. mycoderma, Sacch. cerevisiae, Rhodotorula* sp., *Cephalosporium* sp., *Fusarium* sp.*, Aspergillus*sp., *Penicillium* sp.	Nigeria	Olasupo et al., 2010; Oguntoyinbo et al., 2011
*Kisra*	Sorghum	Thin pancake bread, staple	*Ped. pentosaceus, Lb. confusus, Lb. brevis, Erwinia ananas, Klebsiella pneumoniae, Ent. cloacae, Cand. intermedia, Deb. hansenii, Aspergillus* sp.*, Penicillium* sp.*, Fusarium* sp.*, Rhizopus* sp.	Sudan	Hamad et al., 1997
*Koko*	Maize	Porridge	*Ent. clocae, Acinetobacter* sp.*, Lb. plantarum, Lb. brevis, Sacch. cerevisiae, Cand. mycoderma*	Ghana	Blandino et al., 2003
*Lao-chao*	Rice	Paste, soft, juicy, glutinous dessert	*Rhiz. oryzae, Rhiz. chinensis, Chlamydomucor oryzae, Sacchromycopsis* sp.	China	Blandino et al., 2003
*Mawè*	Maize	Intermediate product used to prepare beverages, porridges	*Lb. fermentum, Lb. reuteri, Lb. brevis, Lb. confusus, Lb. curvatus, Lb. buchneri, Lb. salivarius, Lact. lactis, Ped. pentosaceus, Ped. acidilactici, Leuc. mesenteroides; Candida glabrata, Sacch. cerevisiae, Kluyveromyces marxianus, Clavispora lusitaniae*	Benin, Togo	Greppi et al., 2013a,b
*Mbege*	Maize, sorghum, millet	Submerged	*Sacch. cerevisiae, Schizosaccharomyces pombe, Lb. plantarum, Leuc. mesenteroides*	Tanzania	Odunfa and Oyewole, 1997
*Ogi*	Maize, sorghum, millet	Mild-acidic, viscous, porridge, staple	*Lb. plantarum, Lb. pantheris, Lb. vaccinostercus, Corynebacterium* sp.*, Aerobacter* sp., *Candida krusei, Clavispora lusitaniae, Sacch. cerevisiae, Rhodotorula* sp., *Cephalosporium* sp., *Fusarium* sp.*, Aspergillus* sp., *Penicillium* sp.	Nigeria	Greppi et al., 2013a
*Pito*	Maize, sorghum	Submerged	*Geotrichum candidum, Lactobacillus* sp., *Candida* sp.	West Africa	Odunfa and Oyewole, 1997
*Poto poto*	Maize	Slurry	*Lb. gasseri, Lb. plantarum/paraplantarum, Lb. acidophilus, Lb. delbrueckii, Lb. reuteri, Lb. casei, Bacillus* sp., *Enterococcus* sp., Yeasts	Congo	Abriouel et al., 2006
*Pozol*	Maize	Mild-acidic, thick viscous, porridge, staple	*Strep. bovis, Strep. macedonicus, Lc. lactis, Ent. sulfureus*	Mexico	Díaz-Ruiz et al., 2003
*Puto*	Rice	Steamed cake, breakfast	*Leuc. mesenteroides, Ent. faecalis, Ped. pentosaceus*, Yeasts	Philippines	Steinkraus, 2004
*Rabadi*	Buffalo or cow milk and cereals, pulses	Mild-acidic, thick slurry-like product	*Ped. acidilactici, Bacillus* sp., *Micrococcus* sp., yeasts	India, Pakistan	Gupta et al., 1992
*Selroti*	Rice-wheat flour-milk	Pretzel-like, deep fried bread, staple	*Leuc. mesenteroides, Ent. faecium, Ped. Pentosaceus* and *Lb. curvatus, Sacch. cerevisiae, Sacch. kluyveri, Deb. hansenii, P. burtonii, Zygosaccharomyces rouxii*	India, Nepal, Bhutan	Yonzan and Tamang, 2010, 2013
*Sourdough*	Rye, wheat	Mild-acidic, leavened bread	*Lb. sanfranciscensis, Lb. alimentarius, Lb. buchneri, Lb. casei, Lb. delbrueckii, Lb. fructivorans, Lb. plantarum, Lb. reuteri, Lb. johnsonii, Cand. humili, Issatchenkia orientalis*	America, Europe, Australia	Gänzle et al., 1998; de Vuyst et al., 2009
*Tape Ketan*	Glutinous rice, *Ragi*	Sweet, sour, mild alcoholic, dessert	*Thizopus* sp.*, Chlamydomucor* sp.*, Candida* sp.*, Endomycopsis* sp.*, Saccharomyces* sp.	Indonesia	Steinkraus, 1996
*Togwa*	Cassava, maize, sorghum, millet	Fermented gruel or beverage	*Lb. brevis, Lb. cellobiosus, Lb. fermentum, Lb. plantarum and Ped. pentosaceus, Candida pelliculosa, C. tropicalis, Issatchenkia orientalis, Sacch. cerevisiae*	Tanzania	Mugula et al., 2003
*Tarhana*	Sheep milk, wheat	Mild-acidic, sweet-sour, soup or biscuit	*Lb. bulgaricus, Strep. thermophilus*, yeasts	Cyprus, Greece, Turkey	Sengun et al., 2009
*Uji*	Maize, sorghum, millet, cassava flour	Acidic, sour, porridge, staple	*Leuc. mesenteroides, Lb. plantarum*	Kenya, Uganda, Tanzania	Odunfa and Oyewole, 1997

### Fermented vegetable foods

Perishable and seasonal leafy vegetables, radish, cucumbers including young edible bamboo tender shoots are traditionally fermented into edible products (Table 3). Fermentation of vegetables is mostly dominated by species of *Lactobacillus* and *Pediococcus*, followed by *Leuconostoc, Weissella, Tetragenococcus*, and *Lactococcus* (Chang et al., 2008; Watanabe et al., 2009a). A complete microbial profile of LAB in *kimchi* has been characterized using different molecular identification tools (Shin et al., 2008; Nam et al., 2009; Park et al., 2010; Jung et al., 2011, 2013a). Natural fermentations during production of *sauerkraut*, a fermented cabbage product of Germany, had been studied and a species of LAB were reported. (Johanningsmeier et al., 2007; Plengvidhya et al., 2007). Species of LAB constitute the native population in the Himalayan fermented vegetable products such as *gundruk, sinki, goyang, khalpi*, and *inziangsang* (Karki et al., 1983; Tamang et al., 2005, 2009; Tamang and Tamang, 2007, 2010) and in several naturally fermented bamboo products of India and Nepal (Tamang and Sarkar, 1996; Tamang et al., 2008; Tamang and Tamang, 2009; Jeyaram et al., 2010; Sonar and Halami, 2014).

**Table 3:** **Microorganisms isolated from some common and uncommon fermented vegetable products of the world**.

**Product**	**Substrate/Raw materials**	**Sensory property and nature**	**Microorganisms**	**Country**	**References**
*Burong mustala*	Mustard	Acidic, wet	*Lb. brevis, Ped. cerevisiae*	Philippines	Rhee et al., 2011
*Cucumbers* (fermented)	Cucumbers	Acidic, wet, pickle	*Leuc. mesenteroides, Ped. cerevisiae, Ped. acidilactici, Lb. plantarum, Lb. brevis*	Europe, USA, Canada	Pederson, 1979
*Dha muoi*	Mustard and beet, eggplant	Acidic, wet	*Lb. fermentum, Lb. pentosus, Lb. plantarum, Ped. pentosaceus, Lb. brevis, Lb. paracasei, Lb. pantheris, Ped. acidilactici*	Vietnam	Nguyen et al., 2013a
*Ekung*	Bamboo shoot	Acidic, sour, soft, curry	*Lb. plantarum, Lb. brevis, Lb. casei, Tor. halophilus*	India	Tamang and Tamang, 2009
*Eup*	Bamboo shoot	Acidic, sour, dry, curry	*Lb. plantarum, Lb. fermentum, Lb. brevis, Lb. curvatus, Ped. pentosaceus, Leuc. mesenteroides, Leuc. fallax, Leuc. lactis, Leuc. citreum, Ent. durans*	India	Tamang and Tamang, 2009
*Fu-tsai*	Mustard	Acidic, sour	*Ent. faecalis, Lb. alimentarius, Lb. brevis, Lb. coryniformis, Lb. farciminis, Lb. plantarum, Lb. versmoldensis, Leuc. citreum, Leuc. mesenteroides, Leuc. pseudomesenteroides, Ped. pentosaceus, W. cibaria, W. paramesenteroides*	Taiwan	Chao et al., 2009, 2012
*Goyang*	Wild vegetable	Acidic, sour, wet, soup	*Lb. plantarum, L. brevis, Lc. lactis, Ent. faecium, Ped. pentosaceus, Candida* sp.	India, Nepal	Tamang and Tamang, 2007
*Gundruk*	Leafy vegetable	Acidic, sour, dry, soup, side-dish	*Lb. fermentum, Lb. plantarum, Lb. casei, Lb. casei* subsp. *pseudoplantarum, Ped. pentosaceus*	India, Nepal, Bhutan	Karki et al., 1983; Tamang et al., 2005
*Hirring*	Bamboo shoot tips	Acidic, sour, wet, pickle	*Lb. brevis, Lb. plantarum, Lb. curvatus, Ped. pentosaceus, Leuc. mesenteroides, Leuc. fallax, Leuc. lactis, Leuc. citreum, Ent. durans, Lc. lactis*	India	Tamang and Tamang, 2009
*Hom-dong*	Red onion	Fermented red onion	*Leuc. mesenteroides, Ped. cerevisiae, Lb. plantarum, Lb. fermentum, Lb. buchneri*	Thailand	Phithakpol et al., 1995
*Jiang-gua*	Cucumber	Fermented cucumber, pickle	*Ent. casseliflavus, Leuc. lactis, Leuc. mesenteroides, Lb. pentosus, Lb. plantarum, Lb. paraplantarum, Lc. lactis* subsp. *lactis, W. cibaria, W. hellenica*	Taiwan	Chen et al., 2012
*Jiang-sun*	Bamboo shoot, salt, sugar, *douchi* (fermented soybeans)	Fermented bamboo; side dish	*Lb. plantarum, Ent. faecium, Lc. lactis* subsp. *lactis*	Taiwan	Chen et al., 2010
*Khalpi*	Cucumber	Acidic, sour, wet, pickle	*Lb. brevis, Lb. plantarum, Ped. pentosaceus, Ped. acidilactici, Leuc. fallax*	India, Nepal	Tamang et al., 2005; Tamang and Tamang, 2010
*Kimchi*	Cabbage, green onion, hot pepper, ginger	Acidic, mild-sour, wet, side-dish	*Leuc. mesenteroides, Leuc. citreum, Leuc. gasicomitatum, Leuc. kimchii, Leuc. inhae, W. koreensis, W. kimchii, W. cibaria, Lb. plantarum, Lb. sakei, Lb. delbrueckii, Lb. buchneri, Lb. brevis, Lb. fermentum, Ped. acidilactici, Ped. pentosaceus, Lc. Lactis*, yeasts species of *Candida, Halococcus, Haloterrigena, Kluyveromyces, Lodderomyces, Natrialba, Natronococcus, Pichia, Saccharomyces, Sporisorium* and *Trichosporon*	Korea	Chang et al., 2008; Nam et al., 2009; Jung et al., 2011
*Naw-mai-dong*	Bamboo shoots	Acidic, wet	*Leuc. mesenteroides, Ped. cerevisiae, Lb. plantarum, Lb. brevis, Lb. fermentum, Lb. buchneri*	Thailand	Phithakpol et al., 1995
*Mesu*	Bamboo shoot	Acidic, sour, wet	*Lb. plantarum, Lb. brevis, Lb. curvatus, Leu, citreum, Ped. pentosaceus*	India, Nepal, Bhutan	Tamang et al., 2008
*Oiji*	Cucumber, salt, water	Fermented cucumber	*Leuc. mesenteroides, Lb. brevis, Lb. plantarum, Ped. cerevisiae*	Korea	Alexandraki et al., 2013
*Olives* (fermented)	Olive	Acidic, wet, Salad, side dish	*Leuc. mesenteroides, Ped. pentosaceus; Lb. plantarum Lb. pentosus/Lb. plantarum, Lb. paracollinoides, Lb. vaccinostercus*/*Lb. suebicus* and *Pediococcus* sp. non-lactics (*Gordonia* sp./*Pseudomonas* sp., *Halorubrum orientalis, Halosarcina pallid, Sphingomonas* sp./*Sphingobium* sp./*Sphingopyxis* sp., *Thalassomonas agarivorans*) and yeasts (*Candida* cf. *apicola, Pichia* sp., *Pic. manshurica*/*Pic. galeiformis, Sacch. cerevisiae*)	USA, Spain, Portugal, Peru, Chile	Abriouel et al., 2011
*Pak-gard-dong*	Leafy vegetable, salt, boiled rice	Acidic, wet, side dish	*Lb. plantarum, Lb. brevis, Ped. cerevisiae*	Thailand	Phithakpol et al., 1995
*Pak-sian-dong*	Leaves of *Gynandropis pentaphylla*	Acidic, wet, side dish	*Leuc. mesenteroides, Ped. cerevisiae, Lb. plantarum, Lb. germentum, Lb. buchneri*	Thailand	Phithakpol et al., 1995
*Pao cai*	Cabbage	Sweet and sour rather than spicy, Breakfast	*Lb. pentosus, Lb. plantarum, Lb. brevis, Lb. lactis, Lb. fermentum, and Leuc. mesenteroides*, and *Ped. pentosaceus*	China	Yan et al., 2008
*Sauerkraut*	Cabbage	Acidic, sour, wet, salad, side dish	*Leuc. mesenteroides, Ped. pentosaceus; Lb. brevis, Lb. plantarum, Lb. sakei*	Europe, USA, Canada, Australia	Johanningsmeier et al., 2007
*Sayur asin*	Mustard leaves, cabbage, salt, coconut	Acidic, sour, wet, salad	*Leuc. mesenteroides, Lb. plantarum, Lb. brevis, Lb. confuses, Ped. pentosaceus*.	Indonesia	Puspito and Fleet, 1985
*Soibum*	Bamboo shoot	Acidic, sour, soft, curry	*Lb. plantarum, Lb. brevis, Lb. coryniformis, Lb. delbrueckii, Leuc. fallax, Leuc. Lact. lactis, Leuc. mesenteroides, Ent. durans, Strep. lactis, B. subtilis, B. lichniformis, B. coagulans, B. cereus, B. pumilus, Pseudomonas fluorescens, Saccharomyces* sp., *Torulopsis* sp.	India	Tamang et al., 2008; Jeyaram et al., 2010
*Soidon*	Bamboo shoot tips	Acidic, sour, soft, curry	*Lb. brevis, Lb. plantarum*, uncultured *Lb. acetotolera, Leuc. fallax, Leuc. citreumns, Lc. lactis* subsp. *cremori*s, *Weissella cibaria*, uncultured *W. ghanensis*	India	Tamang et al., 2008; Romi et al., 2015
*Sinki*	Radish tap-root	Acidic, sour, dry, soup, pickle	*Lb. plantarum, Lb. brevis, Lb. casei, Leuc. fallax*	India, Nepal, Bhutan	Tamang and Sarkar, 1993; Tamang et al., 2005
*Suan-cai*	Vegetables	Acidic, sour, wet	*Ped. pentosaceus, Tetragenococcus halophilus*	China	Chen et al., 2006
*Suan-tsai*	Mustard	Acidic, sour, dry	*Ent. faecalis, Lb. alimentarius, Lb. brevis, Lb. coryniformis, Lb. farciminis, Lb. plantarum, Lb. versmoldensis, Leuc. citreum, Leuc. mesenteroides, Leuc. pseudomesenteroides, Ped. pentosaceus, W. cibaria, W. paramesenteroides*	Taiwan	Chao et al., 2009
*Sunki*	Turnip	Acidic, sour, wet	*Lb. plantarum, Lb. fermentum, Lb. delbrueckii, Lb. parabuchneri, Lb. kisonensis, Lb. otakiensis, Lb. rapi, Lb. sunkii*	Japan	Endo et al., 2008; Watanabe et al., 2009a
*Takuanzuke*	Japanese radish, salt, sugar, *Shochu*	Pickle radish	*Lb. plantarum, Lb. brevis, Leuc. mesenteroides, Streptococcus* sp.*, Pediococcus* sp., yeasts	Japan	Alexandraki et al., 2013
*Tuaithur*	Bamboo shoot	Solid, wet, sour, curry	*Lb. plantarum, Lb*. *brevis, Ped. pentosaceou, Lc. lactis, Bacillus circulans, B. firmus, B. sphaericus, B. subtilis*	India	Chakrabarty et al., 2014

### Fermented soybeans and other legumes

Two types of fermented soybean foods are produced: soybean foods fermented by *Bacillus* spp. (mostly *B. subtilis*) with the stickiness characteristic, and soybean foods fermented by filamentous molds, mostly *Aspergillus, Mucor, Rhizopus* (Tamang, 2010b). *Bacillus*-fermented, non-salty and sticky soybean foods are concentrated in an imaginary triangle with three vertices lying each on Japan (*natto*), east Nepal and north-east India (*kinema* and its similar products), and northern Thailand (*thua-nao*), named as “*natto* triangle” (Nakao, 1972) and renamed as “*kinema-natto-thuanao* (KNT)-triangle” (Tamang, 2015b). Within the KNT-triangle-bound countries, *Bacillus*-fermented sticky non-salty soybean foods are consumed such as *natto* of Japan, *chungkokjang* of Korea, *kinema* of India, Nepal and Bhutan, *aakhune, bekang, hawaijar, peruyaan*, and *tungrymbai* of India, *thua nao* of Thailand, *pepok* of Myanmar, and *sieng* of Cambodia and Laos (Nagai and Tamang, 2010; Tamang, 2015b; Table 4). Although, the method of production and culinary practices vary from product to product, plasmids, and phylogenetic analysis of *B. subtilis* showed the similarity among the strains of *B. subtilis* isolated from common sticky fermented soybean foods of Asia (Hara et al., 1986, 1995; Tamang et al., 2002; Meerak et al., 2007) suggesting the common stock of *Bacillus*. Mould-fermented soybean products are *miso* and *shoyu* of Japan, *tempe* of Indonesia, *douchi* and *sufu* of China, and *doenjang* of Korea (Sugawara, 2010). Some common non-soybean fermented legumes (e.g., locust beans) are *bikalga, dawadawa, iru, okpehe, soumbala*, and *dugba* of Africa (Ouoba et al., 2004, 2008, 2010; Amoa-Awua et al., 2006; Azokpota et al., 2006; Oguntoyinbo et al., 2007, 2010; Meerak et al., 2008; Parkouda et al., 2009; Ahaotu et al., 2013), fermented black-grams products such as *dhokla, papad*, and *wari* of India (Nagai and Tamang, 2010), and *maseura* of India and Nepal (Chettri and Tamang, 2008).

**Table 4:** **Microorganisms isolated from some common and uncommon fermented legume (soybeans and non-soybean) products of the world**.

**Product**	**Substrate/Raw material**	**Sensory features and nature**	**Microorganisms**	**Country**	**References**
*Bekang*	Soybean	Alkaline, sticky, paste, curry	*B. subtilis, B. brevis, B. circulans, B. coagulans, B. licheniformis, B. pumilus, B. sphaericus*, and *Lysinibacillus fusiformis*	India	Chettri and Tamang, 2015
*Bhallae*	Black gram	Mild acidic, side dish	*B. subtilis, Candida curvata, C. famata, C. membraneafaciens, C. variovaarai, Cryptococcus humicoius, Deb. hansenii, Geotrichum candidum, Hansenula anomala, H. polymorpha, Kl. marxianus, Lb. fermentum, Leuc. mesenteroides, Ped. membranaefaciens, Rhiz. marina, Sacch. cerevisiae, Ent. faecalis, Trichosporon beigelii, Trichosporon pullulans, Wingea robertsii*	India	Rani and Soni, 2007
*Bikalga*	Roselle (*Hibiscus sabdariffa*)	Condiment	*B. subtilis, B. licheniformis, B. megaterium, B. pumilus*	Burkina Faso	Ouoba et al., 2008
*Chungkokjang* (or *jeonkukjang, cheonggukjang*	Soybean	Alkaline, sticky, soup	*B. subtilis, B. amyloliquefaciens, B. licheniformis, B. cereus, Pantoea agglomerans, Pantoega ananatis, Enterococcus* sp.*, Pseudomonas* sp.*, Rhodococcus* sp.	Korea	Hong et al., 2012; Nam et al., 2012
*Dawadawa*	Locust bean	Alkaline, sticky	*B. pumilus, B. licheniformis, B. subtilis, B. firmus, B. atrophaeus, B. amyloliquefaciens, B. mojavensis, Lysininbacillus sphaericus*.	Ghana, Nigeria	Amoa-Awua et al., 2006; Meerak et al., 2008
*Dhokla*	Bengal gram	Mild acidic, spongy, steamed, snack	*Leuc. mesenteroides, Lb. fermenti, Ent. faecalis, Tor. candida, Tor. pullulans*	India	Blandino et al., 2003
*Douchi*	Soybean	Alkaline, paste	*B. amyloliquefaciens, B. subtilis, Asp. oryzae*	China, Taiwan	Wang et al., 2006; Zhang et al., 2007
*Doenjang*	Soybean	Alkaline, paste, soup	*B. subtilis, B. licheniformis, B. pumilis, Mu. plumbeus, A*sp. *oryzae, Deb. hansenii, Leuc. mesenteroides, Tor. halophilus, Ent. faecium, Lactobacillus* sp.	Korea	Kim et al., 2009; Nam et al., 2011
*Furu*	Soybean curd	Mild acidic	*B. pumilus, B. megaterium, B. stearothermophilus, B. firmus, Staph. hominis*	China	Sumino et al., 2003
*Gochujang*	Soybean, red pepper	Hot-flavored seasoning	*B. velegensis, B. amyloliquefacious, B. subtilis, B. liqueformis*, spcecis of *Oceanobacillus, Zygosaccharomyses, Candida lactis, Zygorouxii, Aspergillus, Penicillium, Rhizopus*	Korea	Shin et al., 2012
*Hawaijar*	Soybean	Alkaline, sticky	*B. subtilis, B. licheniformis, B. amyloliquefaciens, B. cereus, Staph. aureus, Staph. sciuri, Alkaligenes* sp.*, Providencia rettgers, Proteus mirabilis*	India	Jeyaram et al., 2008b; Singh et al., 2014
*Iru*	Locust bean	Alkaline, sticky	*B. subtilis, B*. *pumilus, B*. *licheniformis, B. megaterium, B. fumus, B. atrophaeus, B. amyloliquefaciens, B. mojavensis, Lysininbacillus sphaericus, Staph. saprophyticus*	Nigeria, Benin	Meerak et al., 2008
*Kanjang*	Soybean, *meju*, salt, water	Soya sauce	*Asp*. *oryzae, B. subtilis, B. pumillus, B. citreus, Sarcina mazima, Sacch. rouxii*	Korea	Shin et al., 2012
*Kawal*	Leaves of legume (*Cassia* sp.)	Alkaline, strong flavored, dried balls	*B. subilis, propionibacterium* sp.*, Lb. plantarum, Staph. sciuri*, yeasts	Sudan	Dirar et al., 2006
*Kecap*	Soybean, wheat	Liquid	*Rhiz. oligosporus, Rhiz. oryzae, A*sp. *oryzae, Ped. halophilus, Staphylococcus* sp.*, Candida* sp.*, Debaromyces* sp.*, Sterigmatomyces* sp.	Indonesia	Alexandraki et al., 2013
*Ketjap*	Soybean (black)	Syrup	*A*sp. *oryzae, A*sp. *flavus, Rhiz. oligosporus, Rhiz. arrhizus*	Indonesia	Alexandraki et al., 2013
*Kinda*	Locust bean	Alkaline, sticky	*B. pumilus, B. licheniformis, B. subtilis, B. atrophaeus, B. amyloliquefaciens, B. mojavensis, Lysininbacillus sphaericus*	Sierra Leone	Meerak et al., 2008
*Kinema*	Soybean	Alkaline, sticky; curry	*B. subtilis, B. licheniformis, B. cereus, B. circulans, B. thuringiensis, B*. sp*haericus, Ent. faecium, Cand. parapsilosis, Geotrichum candidum*	India, Nepal, Bhutan	Sarkar et al., 1994; Tamang, 2003
*Maseura*	Black gram	Dry, ball-like, brittle, condiment	*B. subtilis, B. mycoides, B. pumilus, B. laterosporus, Ped. acidilactici, Ped. pentosaceous, Ent. durans, Lb. fermentum, Lb. salivarius, Sacch. cerevisiae, Pic. burtonii, Cand. castellii*	Nepal, India	Chettri and Tamang, 2008
*Meitauza*	Soybean	Liquid	*B. subtilis, A*sp. *oryzae, Rhiz. oligosporus, Mu. meitauza, Actinomucor elegans*	China, Taiwan	Zhu et al., 2008
*Meju*	Soybean	Alkaline, paste	*Asp. flavus, Asp. fumigatus, Asp. niger, Asp. oryzae, Asp. retricus, Asp. spinosa, Asp. terreus, Asp. wentii, Botrytis cineara, Mu. adundans, Mu. circinelloides, Mu. griseocyanus, Mu. hiemalis, Mu. jasseni, Mu. racemosus, Pen. citrinum, Pen. griseopurpureum, Pen. griesotula, Pen. kaupscinskii, Pen. lanosum, Pen. thomii, Pen. turalense, Rhi. chinensis, Rhi. nigricans, Rhi. oryzae, Rhi. Sotronifer; Candida edax, Can. incommenis, Can. utilis Hansenula anomala, Han. capsulata, Han. holstii, Rhodotorula flava, Rho. glutinis, Sacch. exiguus, Sacch. cerevisiae, Sacch. kluyveri, Zygosaccharomyces japonicus, Zyg. rouxii, B. citreus, B. circulans, B. licheniformis, B. megaterium, B. mesentricus, B. subtilis, B. pumilis, Lactobacillus* sp., *Ped. acidilactici*	Korea	Choi et al., 1995
*Miso*	Soybean	Alkaline, paste	*Ped. acidilactici, Leuc. paramesenteroides, Micrococcus halobius, Ped. halophilus, Streptococcus* sp., *Sacch. rouxii, Zygosaccharomyces rouxii, Asp. oryzae*	Japan	Asahara et al., 2006; Sugawara, 2010
*Natto*	Soybean	Alkaline, sticky, breakfast	*B. subtilis (natto)*	Japan	Nagai and Tamang, 2010
*Oncom Hitam* (Black *Oncom*) and *Oncom Merah* (Orange *Oncom*)	Peanut press cake, tapioca, soybean curd starter	Fermented peanut press cake, roasted or fried	*Neurosporaintermedia, N. crassa, N. sitophila* (from red *oncom), Rhi. oligosporus* (from black *oncom*)	Indonesia	Ho, 1986
*Ogiri / Ogili*	Melon Seeds, castor oil seeds, pumpkin bean, sesame		*B. subtilis, B*. *pumilus, B*. *licheniformis, B. megaterium, B. rimus, Pediococcus* sp.*, Staph. saprophyticus, Lb. plantarum*	West, East and Central Africa	Odunfa and Oyewole, 1997
*Okpehe*	Seeds from *Prosopis africana*	Alkaline, sticky	*B. subtilis, B. amyloliquefaciens, B. cereus, B. licheniformis*	Nigeria	Oguntoyinbo et al., 2010
*Soumbala*	Locust bean	Alkaline, sticky	*B. pumilus, B. atrophaeus, B. amyloliquefaciens, B. mojavensis, Lysininbacillus sphaericus. B. subtilis, B. thuringiensis, B. licheniformis, B. cereus, B. badius, B. firmus, B. megaterium, B. mycoides, B. sphaericus, Peanibacillus alvei, Peanibacillus larvae, Brevibacillus latero*sp*orus*	Burkina Faso	Ouoba et al., 2004
*Shoyu*	Soybean	Alkaline, liquid, seasoning	*Asp. oryzae* or *Asp. sojae, Z. rouxii, C. versatilis*	Japan, Korea, China	Sugawara, 2010
*Sufu*	Soybean curd	Mild-acidic, soft	*Actinomucor elenans, Mu. silvatixus, Mu. corticolus, Mu. hiemalis, Mu. praini, Mu. racemosus, Mu. subtilissimus, Rhiz. chinensis*	China, Taiwan	Han et al., 2001; Chao et al., 2008
*Tauco*	Soybean	Alkaline, paste, use as flavoring agent	*Rhiz. oryzae, Rhiz. ologo*sp*orus, Asp*. *oryzae, Zygosaccharomyces soyae, Bacillus* sp., *Ent. hermanniensis, Lb. agilis, Lb. brevis, Lb. buchneri, Lb. crispatus, Lb. curvatus, Lb. delbrueckii, Lb. farciminis, Lb. fermentum, Lb. pantheris, Lb. salivarius, Lb. vaccinostercus, Lc. lactis, Lactococcus* sp., *Leuc. camosum, Leuc. citreum, Leuc. fallax, Leuc. lactis, Leuc. mesenteroides, Leuc. pseudomesenteroides, Ped. acidilactici, Strep. bovis, Strep. macedonicus, W. cibaria, W. confusa, W. paramesenteroides, W. soli*	Indonesia	Winarno et al., 1973
*Tempe*	Soybean	Alkaline, solid, fried cake, breakfast	*Rhiz. oligisporus, Rhiz. arrhizus, Rhiz. oryzae, Rhiz. stolonifer, Asp. niger, Citrobacter freundii, Enterobacter cloacae, K. pneumoniae, K. pneumoniae* subsp. *ozaenae, Pseudomas fluorescens* as vitamin B_12_-producing bacteria, *Lb. fermentum, Lb. lactis, Lb. plantarum, Lb. reuteri*	Indonesia (Origin), The Netherlands, Japan, USA	Feng et al., 2005; Jennessen et al., 2008
*Thua nao*	Soybean	Alkaline, paste, dry, side dish	*B. subtilis, B. pumilus, Lactobacillus* sp.	Thailand	Chunhachart et al., 2006
*Tungrymbai*	Soybean	Alkaline, sticky, curry, soup	*B. subtilis, B. licheniformis, B. pumilus*	India	Chettri and Tamang, 2015
*Ugba*	African oil bean (*Pentaclethra macrophylla*)	Alkaline, flat, glossy, brown in color	*B. subtilis, B*. *pumilus, B*. *licheniformis, Staph. saprophyticus*	Nigeria	Ahaotu et al., 2013
*Wari*	Black gram	Ball-like, brittle, side dish	*B. subtilis, Cand. curvata, Cand. famata, Cand. krusei, Cand. parapsilosis, Cand. vartiovaarai, Cryptococcus humicolus, Deb. hansenii, Deb. tamarii, Geotrichum candidum, Hansenula anomala, Kl. marxianus, Sacch. cerevisiae, Rhiz. lactosa, Ent. faecalis, Wingea robetsii, Trichosporon beigelii*	India	Rani and Soni, 2007
*Yandou*	Soybean	Alkaline, sticky, salted, snack	*B. subtilis*	China	Qin et al., 2013

Species of *Bacillus* have been reported for several Asian fermented soybean foods (Sarkar et al., 2002; Tamang et al., 2002; Tamang, 2003; Park et al., 2005; Inatsu et al., 2006; Choi et al., 2007; Kimura and Itoh, 2007; Shon et al., 2007; Jeyaram et al., 2008b; Dajanta et al., 2009; Kwon et al., 2009; Kubo et al., 2011; Singh et al., 2014; Wongputtisin et al., 2014; Chettri and Tamang, 2015). However, *B. subtilis* is the dominant functional bacterium in Asian fermented soybean foods (Sarkar and Tamang, 1994; Tamang and Nikkuni, 1996; Dajanta et al., 2011). Japanese *natto* is the only *Bacillus*-fermented soybean food now produced by commercial monoculture starter *B. natto*, earlier isolated from naturally fermented *natt*o by Sawamura (Sawamura, 1906). *Ent. Faecium*, as a minor population group, is also present in *kinema* (Sarkar et al., 1994), in *okpehe* (Oguntoyinbo et al., 2007), and in *chungkukjang* (Yoon et al., 2008).

### Fermented root and tuber products

Cassava (*Manihot esculenta*) root is traditionally fermented into staple foods such as *gari* in Nigeria; *fufu* in Togo, Burkina Faso, Benin and Nigeria; *agbelima* in Ghana; *chikawgue* in Zaire; *kivunde* in Tanzania; *kocho* in Ethiopia; and *foo foo* in Nigeria, Benin, Togo, and Ghana, respectively (Franz et al., 2014; Table 5). The initial stage of cassava fermentation is dominated by *Corynebacterium manihot* (Oyewole et al., 2004) with LAB succession (*Lb. acidophilus, Lb. casei, Lb. fermentum, Lb. pentosus, Lb. plantarum*, Oguntoyinbo and Dodd, 2010). Cassava root is also traditionally fermented into sweet dessert known as *tapé* in Indonesia (Tamang, 2010b).

**Table 5:** **Microorganisms isolated from some fermented root crop products of the world**.

**Product**	**Substrate/raw materials**	**Sensory property and nature**	**Microorganisms**	**Country**	**References**
*Chikwangue*	Cassava	Solid state, staple	Species of *Corynebacterium, Bacillus, Lactobacillus, Micrococcus, Pseudomonas, Acinetobacter, Moraxella*	Central Africa, Zaire	Odunfa and Oyewole, 1997
*Cingwada*	Cassava	Solid state	Species of *Corynebacterium, Bacillus., Lactobacillus, Micrococcus*	East and Central Africa	Odunfa and Oyewole, 1997
*Fufu*	Cassava	Submerged, staple	*Bacillus* sp., *Lb. plantarum, Leuc. mesenteroides, Lb. cellobiosus, Lb. brevis; Lb. coprophilus, Lc. lactis; Leuc. lactis, Lb. bulgaricus, Klebsiella* sp., *Leuconostoc* sp., *Corynebacterium* sp.*, Candida* sp.	West Africa	Odunfa and Oyewole, 1997
*Gari*	Cassava	Solid state, staple	*Corynebacterium mannihot, Geotrichum* sp.*, Lb. plantarium, Lb. buchnerri, Leuconsostoc* sp., *Streptococcus* sp.	West and Central Africa	Oyewole et al., 2004
*Lafun /Konkonte*	Cassava	Submerged, staple	*Bacillus* sp., *Klebsiella* sp.*, Candida* sp.*, Aspergillus* sp.*, Leuc. mesenteroides, Corynebacterium manihot, Lb. plantarum, Micrococcus luteus, Geotrichum candidum*	West Africa	Odunfa and Oyewole, 1997
*Tapé*	Cassava	Sweet dessert	*Streptococcus* sp., *Rhizopus* sp., *Saccharomycopsisfibuligera*	Indonesia	Suprianto Ohba et al., 1989
*Tapai Ubi*	Cassava, *Ragi*	Sweet dessert	*Saccharomycopsis fibuligera, Amylomyces rouxii, Mu. circinelloides, Mu. javanicus, Hansenula* spp, *Rhi. arrhizus, Rhi. oryzae, Rhi. chinensis*	Malaysia	Merican and Yeoh, 1989

### Fermented meat products

Fermented meat products are divided into two categories: those made from whole meat pieces or slices such as dried meat and jerky; and those made by chopping or comminuting the meat, usually called sausages (Adams, 2010). Traditionally fermented meat products of many countries have been well-documented (Table 6), such as fermented sausages (Lücke, 2015) and *salami* (Toldra, 2007) of Europe, jerky of America and Africa (Baruzzi et al., 2006), *nham* of Thailand (Chokesajjawatee et al., 2009), and *nem chua* of Vietnam (Nguyen et al., 2013b). The main microbial groups involved in meat fermentation are LAB (Albano et al., 2009; Cocolin et al., 2011; Khanh et al., 2011; Nguyen et al., 2013b), followed by coagulase-negative staphylococci, micrococci and *Enterobacteriaceae* (Cocolin et al., 2011; Marty et al., 2011), and depending on the product, some species of yeasts (Encinas et al., 2000; Tamang and Fleet, 2009), and molds, which may play a role in meat ripening (Lücke, 2015).

**Table 6:** **Microorganisms isolated from some common and uncommon fermented meat products of the world**.

**Product**	**Substrate/Raw materials**	**Sensory property and nature**	**Microorganisms**	**Country**	**References**
*Alheira*	Pork or beef, bread chopped fat, spices, salt	Dry/Semi-dry, sausage	*Lb. plantarum, Lb. paraplantarum, Lb. brevis, Lb. rhamnosus, Lb. sakei, Lb. zeae, Lb. paracasei, Ent. faecalis, Ent. faecium, Leuc. mesenteroides, Ped. pentosaceus, Ped. acidilactici, W. cibaria, W. viridescens*	Portugal	Albano et al., 2009
*Androlla*	Pork, coarse chopped, spices, salt	Dry, pork sausage	*Lb. sake, Lb. curvatus, Lb. plantarum*	Spain	Garcia-Fontan et al., 2007
*Arjia*	Large intestine of chevon	Sausage, curry	*Ent. faecalis, Ent. faecium, Ent. hirae, Leuc. citreum, Leuc. mesenteroides, Ped. pentosaceus, Weissella cibaria*	India, Nepal	Oki et al., 2011
*Chartayshya*	Chevon	Dried, smoked meat, curry	*Ent. faecalis, Ent. faecium, Ent. hirae, Leuc. citreum, Leuc. mesenteroides, Ped. pentosaceus, Weissella cibaria*	India	Oki et al., 2012
*Chorizo*	Pork	Dry, coarse chopped, spices, salt; sausage	*Lb. sake, Lb. curvatus, Lb. plantarum*	Spain	Garcia-Fontan et al., 2007
*Kargyong*	Yak, beef, pork, crushed garlic, ginger, salt	Sausage like meat product, curry	*Lb. sakei, Lb. divergens, Lb. carnis, Lb. sanfranciscensis, Lb. curvatus, Leuc. mesenteroides, Ent. faecium, B. subtilis, B. mycoides, B. thuringiensis, Staph. aureus, Micrococcus* sp., *Deb. hansenii, Pic. anomala*	India	Rai et al., 2010
*Nham (Musom)*	Pork meat, pork skin, salt, rice, garlic	Fermented pork	*Ped. cerevisiae, Lb. plantarum, Lb. brevis*	Thailand	Chokesajjawatee et al., 2009
*Nem-chua*	Pork, salt, cooked rice	Fermented sausage	*Lb. pentosus, Lb. plantarum, Lb. brevis, Lb. paracasei, Lb. fermentum, Lb. acidipiscis, Lb. farciminis, Lb. rossiae, Lb. fuchuensis, Lb. namurensis, Lc. lactis, Leuc. citreum, Leuc. fallax, Ped. acidilactici, Ped. pentosaceus, Ped. stilesii, Weissella cibaria, W. paramesenteroides*	Vietnam	Nguyen et al., 2011
*Pastirma*	Chopped beef meat with lamb fat, heavily seasoned	Dry/semi-dry, sausage	*Lb. plantarum, Lb. sake, Pediococcus, Micrococcus, Staph. xylosus, Staph. carnosus*	Turkey, Iraq	Aksu et al., 2005
*Peperoni*	Pork, beef	Dried meat, smoked, sausage	Species of LAB, *Micrococcus* spp.	Europe, America, Australia	Adams, 2010
*Sai-krok-prieo*	Pork, rice, garlic, salt	Fermented sausage	*Lb. plantarum, Lb. salivarius, Ped. pentosacuns*	Thailand	Adams, 2010
*Salchichon*	Pork or beef meat, fat, NaCl, spices	Dry, sausage	Species of LAB, *Staph*. spp., *Micrococcus* spp., enterobacteriaceae, molds	Spain	Fernandez-Lopez et al., 2008
*Salsiccia*	Chopped pork meat, spices, NaCl	Dry/ semi-dry, sausage	Species of LAB, *Staph*. spp., *Micrococcus* spp., enterobacteriaceae, yeast	Italy	Parente et al., 2001a,b
*Soppressata*	Chopped lean pork meat, NaCl and spices	Dry/ semi-dry, sausage	Species of LAB, *Staph*. spp., *Micrococcus* spp., enterobacteriaceae, yeast	Italy	Parente et al., 1994
*Sucuk*	Chopped meat, pork or beef, curing salts and various spices	Dry, sausage	Species of LAB, *Staph*. spp., *Micrococcus* spp., enterobacteriaceae	Turkey	Genccelep et al., 2008
*Suka ko masu*	Goat, buffalo meat, turmeric powder, mustard oil, salt	Dried or smoked meat, curry	*Lb. carnis, Ent. faecium, Lb. plantarum, B. subtilis, B. mycoides, B. thuringiensis, Staph. aureus, Micrococcus* sp.*, Debaromyces hansenii, Pic. burtonii*	India	Rai et al., 2010
*Tocino*	Pork, salt, sugar, potassium nitrate	Fermented cured pork	*Ped. cerevisiae, Lb. brevis, Leuc. mesenteroides*	Philippines	Alexandraki et al., 2013

### Fermented fish products

Preservation of fish through fermentation, sun/smoke drying and salting (Table 7) is traditionally practiced by people living nearby coastal regions, lakes, and rivers and is consumed as seasoning, condiments, and side dishes (Salampessy et al., 2010). Several species of bacteria and yeasts have been reported from fermented and traditionally preserved fish products of the world (Kobayashi et al., 2000a,b,c; Wu et al., 2000; Thapa et al., 2004, 2006, 2007; Saithong et al., 2010; Hwanhlem et al., 2011; Romi et al., 2015).

**Table 7:** **Microorganisms isolated from some common and uncommon fermented fish products of the world**.

**Product**	**Substrate/raw materials**	**Sensory property and nature**	**Microorganisms**	**Country**	**References**
*Balao-balao (Burong Hipon Tagbilao)*	Shrimp, rice, salt	Fermented rice shrimp, condiment	*Leuc. mesenteroides, Ped. cerevisiae, Lb. plantarum, Lb. brevis, Ent. faecalis*	Philippines	Alexandraki et al., 2013
*Belacan (Blacan)*	Shrimp, salt	Paste, condiment	*Bacillus, Pediococcus, Lactobacillus, Micrococcus, Sarcina, Clostridium, Brevibacterium, Flavobacterium, Corynebacteria*	Malaysia	Salampessy et al., 2010
*Bakasang*	Fish, shrimp	Paste, condiment	*Pseudomonas, Enterobacter, Moraxella, Micrococcus, Streptococcus, Lactobacillus, Pseudomonas, Moraxella, Staphylococcus, Pediococcus* spp.	Indonesia	Ijong and Ohta, 1996
*Burong Bangus*	Milkfish, rice, salt, vinegar	Fermented milkfish, sauce	*Leuc. mesenteroides, Lb. plantarum, W. confusus*	Philippines	Dalmacio et al., 2011
*Burong Isda*	Fish, rice, salt	Fermented fish, sauce	*Leuc. mesenteroides, Ped. cerevisiae, Lb. plantarum, Strep. faecalis, Micrococcus* sp.	Philippines	Sakai et al., 1983
*Budu*	Marine fishes, salt, sugar	Muslim sauce, fish sauce	*Ped. halophilus, Staph. aureus, Staph. epidermidis, B. subtilis, B. laterosporus, Proteus* sp.*, Micrococcus* sp.*, Sarcina* sp.*, Corynebacterium* sp.	Thailand, Malayasia	Phithakpol et al., 1995
*Gnuchi*	Fish (*Schizothorax richardsonii*), salt, turmeric powder	Eat as curry	*Lb. plantarum, Lact. lactis, Leuc. mesenteroides, Ent. faecium, Ent. faecalis, Ped. pentosaceus, Cand. chiropterorum, Cand. bombicola, Saccharomycopsis* sp.	India	Tamang et al., 2012
*Gulbi*	Shell-fish	Salted and dried, side dish	*Bacillus licheniformis, Staphylococcus* sp., *Aspergillus* sp., *Candida* sp.	Korea	Kim et al., 1993
*Hentak*	Finger sized fish (*Esomus danricus*)	Condiment	*Lact. lactis, Lb. plantarum, Lb. fructosus, Lb. amylophilus, Lb. coryniformis, Ent. faecium, B. subtilis, B. pumilus, Micrococcus* sp.*, Candida* sp.*, Saccharomycopsis* sp.	India	Thapa et al., 2004
*Hoi-malaeng pu-dong*	Mussel (*Mytilus smaragdinus*), salt	Fermented mussel	*Ped. halophilus, Staph. aureus, Staph. epidermidis*	Thailand	Phithakpol et al., 1995
*Ika-Shiokara*	Squid, salt	Fermented squid	*Micrococcus* sp.*, Staphylococcus* sp.*, Debaryomyces* sp.	Japan	Alexandraki et al., 2013
*Jeotkal*	Fish	High-salt fermented, staple	LAB, halophilicFirmicutes including *Staphylococcus, Salimicrobium*, and *Alkalibacillus*. Also *Halanaerobium* and halophilic archaea.	Korea	Guan et al., 2011; Jung et al., 2013b
*Karati, Bordia, Lashim*	Fish (*Gudushia chapra, Pseudeutropius atherinoides, Cirrhinus reba*), salt	Dried, salted, side dish	*Lact. lactis, Leuc. mesenteroides, Lb. plantarum, B. subtilis, B. pumilus, Candida* sp.	India	Thapa et al., 2007
*Kusaya*	Horse mackerel, salt	Fermented dried fish	*Corynebacterium kusaya, Spirillum* sp.*, C. bifermentans, Penicillium* sp.	Japan	Alexandraki et al., 2013
*Myulchijeot*	Small sardine, salt	Fermented sardine	*Ped. cerevisiae, Staphylococcus* sp.*, Bacillus* sp.*, Micrococcus* sp.	Korea	Alexandraki et al., 2013
*Narezushi*	Sea water fish, cooked millet, salt	Fermented fish-rice	*Leuc. mesenteroides, Lb. plantarum*	Japan	Alexandraki et al., 2013
*Nam pla (Nampla-dee, Nampla-sod)*	*Solephorus* sp., *Ristelliger* sp. *Cirrhinus* sp., water, salt	Fish sauce	Species of *Micrococcus*.*, Pediococcus, Staphylococcus*.*, Streptococcus*.*, Sarcina*.*, Bacillus*.*, Lactobacillus, Corynebacterium, Pseudomonas, Halococcus, Halobacterium*	Thailand	Saisithi, 1987
*Ngari*	Fish (*Puntius sophore*), salt	Fermented fish	*Lact. lactis, Lb. plantarum, Lb. pobuzihii, Lb. fructosus, Lb. amylophilus, Lb. coryniformis, Ent. faecium, B. subtilis, B. pumilus, B indicu, s Micrococcus* sp.*, Staphy. cohnii* subsp. *cohnii, Staphy. carnosus, Tetragenococcus halophilus* subsp. *flandriensis, Clostridium irregular, Azorhizobium caulinodans, Candida* sp.*, Saccharomycopsis* sp.	India	Thapa et al., 2004; Devi et al., 2015
*Nuoc mam*	Marne fish	Fish sauce, condiment	*Bacillus* sp., *Pseudomonas* sp., *Micrococcus* sp., *Staphylococcus* sp., *Halococcus* sp., *Halobacterium salinarium, H. cutirubrum*	Vietnam	Lopetcharat et al., 2001
*Patis*	*Stolephorus* sp., *Clupea* sp., *Decapterus* sp., *Leionathus* sp., salt	Fish sauce	*Ped. halophilus, Micrococcus* sp.*, Halobacterium* sp.*, Halococcus* sp.*, Bacillus* sp.	Philippines, Indonesia	Steinkraus, 1996
*Pla-paeng-daeng*	Marine fish, red molds rice (*Ang-kak*), salt	Red fermented fish	*Pediococcus* sp.*, Ped. halophilus, Staph. aureus, Staph. epidermidis*,	Thailand	Phithakpol et al., 1995
*Pla-som (Pla-khao-sug)*	Marine fish, salt, boiled rice, garlic	Fermented fish, condiment	*Ped. cerevisiae, Lb. brevis, Staphylococcus* sp.*, Bacillus* sp.	Thailand	Saithong et al., 2010
*Saeoo Jeot (Jeotkal)*	Shrimp (*Acetes chinensis*), salt	Fermented shrimp	*Halobacterium* sp.*, Pediococcus* sp.	Korea	Guan et al., 2011
*Shidal*	*Puntis*	Semi-fermented, unsalted product; 4–6 months fermentation; curry/pickle	*Staphy. aureus, Micrococcus* spp., *Bacillus* spp., *E. coli*)	India, Bangladesh	Muzaddadi, 2015
*Shottsuru*	Anchovy, opossum shrimp, salt	Fish sauce, condiment	*Halobacterium* sp.*, Aerococcus viridians (Ped. homari)*, halotolerant and halophilic yeasts	Japan	Itoh et al., 1993
*Sidra*	Fish (*Punitus sarana*)	Dried fish, curry	*Lact. lactis, Lb. plantarum, Leuc. mesenteroides, Ent. faecium, Ent. facalis, Ped. pentosaceus, W. confusa, Cand. chiropterorum, Cand. bombicola, Saccharomycopsis* sp.	India	Thapa et al., 2006
*Sikhae*	Sea water fish, cooked millet, salt	Fermented fish-rice, sauce	*Leuc. mesenteroides, Lb. plantarum*	Korea	Lee, 1993
*Suka ko maacha*	River fish (*Schizothorax richardsoni*), salt, turmeric powder	Smoked, dried, curry	*Lact. lactis, Lb. plantarum, Leuc. mesenteroides, Ent. faecium, Ent. faecalis, Ped. pentosaceus, Cand. chiropterorum, Cand. bombicola, Saccharomycopsis* sp.	India	Thapa et al., 2006
*Sukuti*	Fish (*Harpodon nehereus*)	Pickle, soup and curry	*Lact. lactis, Lb. plantarum, Leuc. mesenteroides, Ent. faecium, Ent. faecalis, Ped. pentosaceus, Cand. chiropterorum, Cand. bombicola, Saccharomycopsis* sp.	India	Thapa et al., 2006
*Surströmming*	Fish	Fermented herrings	*Haloanaerobium praevalens*	Sweden	Kobayashi et al., 2000a
*Tungtap*	Fish	Fermented fish, paste, pickle	*Lc. lactis* subsp. *cremoris, Lc. plantarumEnt. faecium, Lb. fructosus, Lb. amylophilus, Lb. corynifomis* subsp. *torquens, Lb. plantarum, Lb. puhozi, B. subtilis, B. pumilus, Micrococcus*, yeasts-species of *Candida, Saccharomycopsis*	India	Thapa et al., 2004; Rapsang et al., 2011

### Miscellaneous fermented products

Vinegar is one of the most popular condiments in the world and is prepared from sugar or ethanol containing substrates and hydrolyzed starchy materials by aerobic conversion to acetic acid (Solieri and Giudici, 2008). *Acetobacter aceti* subsp. *aceti, Acetobacter pasteurianus, Acetobacter polyxygenes, Acetobacter xylinum, Acetobacter malorum, Acetobacter pomorum* dominate during vinegar production (Haruta et al., 2006), while yeast species such as *Candida lactis-condensi, Candida stellata, Hanseniaspora valbyensis, Hanseniaspora osmophila, Saccharomycodes ludwigii, Sacch. cerevisiae, Zygosaccharomyces bailii, Zygosaccharomyces bisporus, Zygosaccharomyces lentus, Zygosaccharomyces mellis, Zygosaccharomyces Pseudorouxii*, and *Zygosaccharomyces Rouxii* have also been reported (Sengun and Karabiyikli, 2011).

Though normal black tea is consumed everywhere, some ethnic Asian communities enjoy special fermented teas such as *miang* of Thailand (Tanasupawat et al., 2007) and *puer* tea, *fuzhuan brick*, and *kombucha* of China (Mo et al., 2008). *Aspergillus niger* is the predominant fungus in *puer* tea while *Blastobotrys adeninivorans, Asp. glaucus*, species of *Penicillium, Rhizopus*, and *Saccharomyces* and the bacterial species *Actinoplanes* and *Streptomyces* are isolated (Jeng et al., 2007; Abe et al., 2008). *Brettanomyces bruxellensis, Candida stellata, Rhodotorula mucilaginosa, Saccharomyces* spp.*, Schizosaccharomyces pombe, Torulaspora delbrueckii, Zygosaccharomyces bailii, Zygosaccharomyces bisporus, Zygosaccharomyces kombuchaensis*, and *Zygosaccharomyces microellipsoides* are also isolated from *kombucha* (Kurtzman et al., 2001; Teoh et al., 2004). Major bacterial genera present in *kombucha* are *Gluconacetobacter*. However, Marsh et al. (2014) reported the predominance of *Lactobacillus, Acetobacter*, and *Zygosaccharomyces*. *Lb. thailandensis, Lb. camelliae, Lb. plantarum, Lb. pentosus, Lb*. *vaccinostercus, Lb. pantheris, Lb. fermentum, Lb. suebicus, Ped. siamensis, Ent. casseliflavus* and *Ent. camelliae* in the fermentation of *miang* production (Sukontasing et al., 2007; Tanasupawat et al., 2007). Species of *Aspergillus, Penicillium*, and *Eurotium* are major fungi for fermentation of *fuzhuan brick* tea (Mo et al., 2008).

*Nata* or bacterial cellulose produced by *Acetobacter xylinum* is a delicacy of the Philippines, eaten as candy (Chinte-Sanchez, 2008; Jagannath et al., 2010; Adams, 2014). Two types of *nata* are well-known: *nata de piña*, produced on the juice from pineapple trimmings, and *nata de coco*, produced on coconut water or coconut skim milk (Adams, 2014). Bacterial cellulose has significant potential as a food ingredient in view of its high purity, *in situ* change of flavor and color, and having the ability to form various shapes and textures (Shi et al., 2014).

Chocolate is a product of cocoa bean fermentation where *Lb. fermentum* and *Acetobacter pasteurianus* are reported as the predominating bacterial species (Lefeber et al., 2010; Papalexandratou et al., 2011). Diverse LAB species appear to be typically associated with the fermentation of cocoa beans in Ghana, which include *Lb. ghanensis* (Nielsen et al., 2007), *Weissella ghanensis* (de Bruyne et al., 2008a), *Lb. cacaonum*, and *Lb. fabifermentans* (de Bruyne et al., 2009), and *Weissella fabaria* (de Bruyne et al., 2010). *Fructobacillus pseudoficulneus, Lb. plantarum, Acetobacter senegalensis*, and the enterobacteria *Tatumella ptyseos* and *Tatumella citrea* are among the prevailing species during the initial phase of cocoa fermentations (Papalexandratou et al., 2011). Yeasts involved during spontaneous cocoa fermentation are *Hanseniaspora uvarum, Hanseniaspora quilliermundii, Issatchenkia orientalis* (*Candida krusei*), *Pichia membranifaciens, Sacch. Cerevisiae*, and *Kluyveromyces* species for flavor development (Schillinger et al., 2010).

*Pidan* is a preserved egg prepared from alkali-treated fresh duck eggs and is consumed by the Chinese, and has a strong hydrogen sulfide and ammonia smell (Ganasen and Benjakul, 2010). The main alkaline chemical reagent used for making *pidan* is sodium hydroxide, which is produced by the reaction of sodium carbonate, water, and calcium oxide of pickle or coating mud. *B. cereus, B*. *macerans, Staph. cohnii, Staph. epidermidis, Staph. Haemolyticus*, and *Staph. warneri* are predominant in *pidan* (Wang and Fung, 1996).

### Amylolytic starters

Traditional way of culturing the essential microorganisms (consortia of filamentous molds, amylolytic, and alcohol-producing yeasts and LAB) with rice or wheat as the base in the form of dry, flattened or round balls, for production of alcoholic beverages is a remarkable discovery in the food history of Asian people, which is exclusively practiced in South-East Asia including the Himalayan regions of India, Nepal, Bhutan, and China (Tibet; Hesseltine, 1983; Tamang, 2010a). Around 1–2% of previously prepared amylolytic starters are inoculated into the dough, and mixed cultures are allowed to develop for a short time, then dried, and used to make either alcohol or fermented foods from starchy materials (Tamang et al., 1996). Asian amylolytic starters have different vernacular names such as *marcha* in India and Nepal; *hamei, humao, phab* in India; *mana* and *manapu* of Nepal; *men* in Vietnam; *ragi* in Indonesia; *bubod* in Philippines; *chiu/chu* in China and Taiwan; *loogpang* in Thailand; *mae/dombae/buh/puh* in Cambodia; and *nuruk* in Korea (Hesseltine and Kurtzman, 1990; Nikkuni et al., 1996; Sujaya et al., 2004; Thanh et al., 2008; Yamamoto and Matsumoto, 2011; Tamang et al., 2012).

Microbial profiles of amylolytic starters of India, Nepal, and Bhutan are filamentous molds like, *Mucor circinelloides* forma *circinelloides, Mucor hiemalis, Rhi. chinensis*, and *Rhi. stolonifer* variety *lyococcus* (Tamang et al., 1988); yeasts like *Sacch. cerevisiae, Sacch. bayanus, Saccharomycopsis* (*Sm*.) *fibuligera, Sm. capsularis, Pichia anomala, Pic. burtonii*, and *Candida glabrata*; (Tamang and Sarkar, 1995; Shrestha et al., 2002; Tsuyoshi et al., 2005; Tamang et al., 2007; Jeyaram et al., 2008a, 2011; Chakrabarty et al., 2014); and species of LAB namely *Ped. pentosaceus, Lb. bifermentans*, and *Lb. brevis* (Hesseltine and Ray, 1988; Tamang and Sarkar, 1995; Tamang et al., 2007; Chakrabarty et al., 2014). A diversity of yeasts (*Candida tropicalis, Clavispora lusitaniae, Pichia anomala, Pichia ranongensis, Saccharomycopsis fibuligera, Sacch. cerevisiae, Issatchenkia* sp.); filamentous molds (*Absidia corymbifera, Amylomyces rouxii, Botryobasidium subcoronatum, Rhizopus oryzae, Rhi. microsporus, Xeromyces bisporus*); LAB (*Ped. pentosaceus, Lb. plantarum, Lb. brevis, Weissella confusa, Weissella paramesenteroides*); amylase-producing bacilli (*Bacillus subtilis, B. circulans, B. amyloliquefaciens, B. sporothermodurans*); and acetic acid bacteria (*Acetobacter orientalis, A. pasteurianus*) is present in *men*, a starter culture of Vietnam (Dung et al., 2006, 2007; Thanh et al., 2008).

A combination of *Asp. oryzae* and *Asp. sojae* is used in *koji* in Japan to produce alcoholic beverages including *saké* (Zhu and Trampe, 2013). *Koji* (Chinese *chu, shi*, or *qu*) also produces amylases that convert starch to fermentable sugars, which are then used for the second stage yeast fermentation to make non-alcoholic fermented soybean *miso* and *shoyu* (Sugawara, 2010). *Asp. awamori, Asp. kawachii, Asp. oryzae, Asp. shirousamii*, and *Asp. sojae* have been widely used as the starter in preparation of *koji* for production of *miso, saké, shoyu, shochu* (Suganuma et al., 2007).

### Alcoholic beverages

Tamang (2010c) classified alcoholic beverages of the world into 10 types:

1. Non-distilled and unfiltered alcoholic beverages produced by amylolytic starters e.g., kodo ko jaanr (fermented finger millets; Thapa and Tamang, 2004) and bhaati jaanr (fermented rice) of India and Nepal (Tamang and Thapa, 2006), makgeolli (fermented rice) of Korea (Jung et al., 2012).
2. Non-distilled and filtered alcoholic beverages produced by amylolytic starters e.g., saké of Japan (Kotaka et al., 2008).
3. Distilled alcoholic beverages produced by amylolytic starter e.g., shochu of Japan, and soju of Korea (Steinkraus, 1996).
4. Alcoholic beverages produced by involvement of amylase in human saliva e.g., chicha of Peru (Vallejo et al., 2013).
5. Alcoholic beverages produced by mono- (single-strain) fermentation e.g., beer (Kurtzman and Robnett, 2003).
6. Alcoholic beverages produced from honey e.g., tej of Ethiopia (Bahiru et al., 2006).
7. Alcoholic beverages produced from plant parts e.g., pulque of Mexico (Lappe-Oliveras et al., 2008), toddy of India (Shamala and Sreekantiah, 1988), and kanji of India (Kingston et al., 2010).
8. Alcoholic beverages produced by malting (germination) e.g., sorghum (“Bantu”) beer of South Africa (Kutyauripo et al., 2009), pito of Nigeria, and Ghana (Kolawole et al., 2013), and tchoukoutou of Benin (Greppi et al., 2013a).
9. Alcoholic beverages prepared from fruits without distillation e.g., wine, cider.
10. Distilled alcoholic beverages prepared from fruits and cereals e.g., whisky and brandy.

### Non-distilled mild-alcoholic food beverages produced by amylolytic starters

The biological process of liquefaction and saccharification of cereal starch by filamentous molds and yeasts, supplemented by amylolytic starters, under solid-state fermentation is one of the two major stages of production of alcoholic beverages in Asia (Tamang, 2010c). These alcoholic beverages are mostly considered as food beverage and eaten as staple food with high calorie in many parts of Asia, e.g., *kodo ko jaanr* of the Himalayan regions in India, Nepal, Bhutan, and China (Tibet) with 5% alcohol content (Thapa and Tamang, 2004). Saccharifying activities are mostly shown by *Rhizopus* spp. and *Sm. fibuligera* whereas, liquefying activities are shown by *Sm. fibuligera* and *Sacch. cerevisiae* (Thapa and Tamang, 2006). *Rhizopus, Amylomyces, Torulopsis*, and *Hansenula* are present in *lao-chao*, a popular ethnic fermented rice beverage of China (Wei and Jong, 1983). During fermentation of Korean *makgeolli* (prepared from rice by amylolytic starter *nuruk*), the proportion of the *Saccharomycetaceae* family increases significantly and the major bacterial phylum of the samples shifts from γ*-Proteobacteria* to *Firmicutes* (Jung et al., 2012).

### Non-distilled and filtered alcoholic beverages produced by amylolytic starters

Alcoholic beverages produced by amylolytic starter (*koji*) are not distilled but the extract of fermented cereals is filtered into clarified high alcohol-content liquor, like in *sake*, which is a national drink of Japan containing 15–20% alcohol (Tamang, 2010c). Improved strains of *Asp. oryzae* are used for *saké* production in industrial scale (Kotaka et al., 2008; Hirasawa et al., 2009).

### Distilled alcoholic beverages produced by amylolytic starters

This category of alcoholic drinks is the clear distillate of high alcohol content prepared as drink from fermented cereal beverages by using amylolytic starters. *Raksi* is an ethnic alcoholic (22–27% v/v) drink of the Himalayas with aromatic characteristic, and distilled from the traditionally fermented cereal beverages (Kozaki et al., 2000).

### Alcoholic beverages produced by human saliva

*Chicha* is a unique ethnic fermented alcoholic (2–12% v/v) beverage of Andes Indian race of South America mostly in Peru, prepared from maize by human salivation process (Hayashida, 2008). *Sacch. cerevisiae, Sacch. apiculata, Sacch. pastorianus*, species of *Lactobacillus* and *Acetobacter* are present in *chicha* (Escobar et al., 1996). *Sacch. cerevisiae* was isolated from *chicha* and identified using MALDI-TOF (Vallejo et al., 2013). Species of *Lactobacillus, Bacillus, Leuconostoc, Enterococcus, Streptomyces, Enterobacter, Acinetobacter, Escherichia, Cronobacter, Klebsiella, Bifidobacterium*, and *Propioniobacterium* have been reported from *chicha* of Brazil (Puerari et al., 2015).

### Alcoholic beverages produced from honey

Some alcoholic beverages are produced from honey e.g., *tej* of Ethiopia. It is a yellow, sweet, effervescent and cloudy alcoholic (7–14% v/v) beverage (Steinkraus, 1996). *Sacch. cerevisiae, Kluyvermyces bulgaricus, Debaromyces phaffi*, and *Kl. veronae*, and LAB species of *Lactobacillus, Streptococcus, Leuconostoc*, and *Pediococcus* are responsible for *tej* fermentation (Bahiru et al., 2006).

### Alcoholic beverages produced from plant parts

*Pulque* is one of the oldest alcoholic beverages prepared from juices of the cactus (*Agave*) plant of Mexico (Steinkraus, 2002). Bacteria present during the fermentation of *pulque* were LAB (*Lc. lactis* subsp. *lactis, Lb. acetotolerans, Lb. acidophilus, Lb. hilgardii, Lb. kefir, Lb. plantarum, Leuc. citreum, Leuc. kimchi, Leuc. mesenteroides, Leuc. pseudomesenteroides*), the γ-Proteobacteria (*Erwinia rhapontici, Enterobacter* spp., and *Acinetobacter radioresistens*, several α-Proteobacteria), *Zymomonas mobilis, Acetobacter malorum, A. pomorium, Microbacterium arborescens, Flavobacterium johnsoniae, Gluconobacter oxydans*, and *Hafnia alvei* (Escalante et al., 2004, 2008). Yeasts isolated from *pulque* are *Saccharomyces (Sacch. bayanus, Sacch. cerevisiae, Sacch. paradoxus*) and non-*Saccharomyces (Candida* spp.*, C. parapsilosis, Clavispora lusitaniae, Hanseniaspora uvarum, Kl. lactis, Kl. marxianus, Pichia membranifaciens, Pichia* spp., *Torulaspora delbrueckii*; Lappe-Oliveras et al., 2008).

Depending on the region, traditional alcoholic drinks prepared from palm juice called “palm wine” are known by various names, e.g., *toddy* or *tari* in India, *mu, bandji, ogogoro, nsafufuo, nsamba, mnazi, yongo, taberna, tua*, or *tubak* in West Africa and South America (Ouoba et al., 2012). Microorganisms that are responsible for *toddy* fermentation are *Sacch. cerevisiae, Schizosaccharomyces pombe, Acetobacter aceti, A. rancens, A. suboxydans, Leuc. dextranicum (mesenteroides), Micrococcus* sp., *Pediococcus* sp., *Bacillus* sp., and *Sarcina* sp. (Shamala and Sreekantiah, 1988).

*Kanji* is an ethnic Indian strong-flavored mild alcoholic beverage prepared from beet-root and carrot by natural fermentation (Batra and Millner, 1974). *Hansenlu anomala, Candida guilliermondii, C. tropicalis, Geotrichium candidum, Leuc. mesenteroides, Pediococcus* sp., *Lb. paraplantarum*, and *Lb. pentosus* are present in *kanji* (Batra and Millner, 1976; Kingston et al., 2010).

### Alcoholic beverages produced by malting or germination

*Bantu* beer or sorghum beer of Bantu tribes of South Africa is an alcoholic beverage produced by malting or germination process (Taylor, 2003). Malted beer is common in Africa with different names e.g., as *bushera* or *muramba* in Uganda, *chibuku* in Zimbabwe, *dolo, burkutu*, and *pito* in West Africa and *ikigage* in Rwanda (Myuanja et al., 2003; Sawadogo-Lingani et al., 2007; Lyumugabe et al., 2012). Sorghum (*Sorghum caffrorum* or *S. vulgare*) is malted (Kutyauripo et al., 2009), characterized by a two-stage (lactic followed by alcoholic) fermentation, with *Lb. fermentum* as the dominating LAB species (Sawadogo-Lingani et al., 2007).

### Alcoholic beverages produced from fruits without distillation

The most common example of alcoholic beverages produced from fruits without distillation is wine, which is initiated by the growth of various species of *Saccharomyces* and non-*Saccharomyces* (so-called “wild”) yeasts (e.g., *Candida colliculosa, C. stellata, Hanseniaspora uvarum, Kloeckera apiculata, Kl. thermotolerans, Torulaspora delbrueckii, Metschnikowia pulcherrima*; Pretorius, 2000; Moreira et al., 2005; Sun et al., 2014; Walker, 2014). *Candida* sp. and *Cladosporium* sp. were isolated from fermenting white wine using mCOLD-PCR-DGGE, but had not been detected by conventional PCR (Takahashi et al., 2014). *Sacch. cerevisiae* strains developed during wine fermentations play an active role in developing the characteristics of a wine (Capece et al., 2013). *Saccharomyces Genome Database* (SGD; www.yeastgenome.org) provides free of charge access or links to comprehensive datasets comprising genomic, transcriptomic, proteomic and metabolomic information (Pretorius et al., 2015).

## Conclusions

Every community in the world has distinct food culture including fermented foods and alcoholic beverages, symbolizing the heritage and socio-cultural aspects of the ethnicity. The word “culture” denotes food habits of ethnicity; another meaning for the same word “culture” is a cluster of microbial cells or inoculum, an essential biota for fermentation, often used in the microbiology. The diversity of functional microorganisms ranges from filamentous molds to enzyme-producing and alcohol-producing yeasts, and from Gram-positive to a few Gram-negative bacteria, while even *Archaea* has been ascribed roles in some fermented foods and alcoholic beverages. However, consumption of lesser known and uncommon ethnic fermented foods is declining due to the change in lifestyles that is shifting from cultural food habits to commercial foodstuffs and fast foods, drastically affecting traditional culinary practices, and also due to the climate change in some environments such as the Sahel region in Africa and the vast areas adjacent to the Gobi desert in Asia.

## Author contributions

JT: contributed 50% of review works. WH, contributed 25% of review. KW contributed 25% of review.

### Conflict of interest statement

The authors declare that the research was conducted in the absence of any commercial or financial relationships that could be construed as a potential conflict of interest.
